# A Case of Rheumatoid Arthritis-Associated Interstitial Lung Disease - An Unfortunate Complication of Untreated Rheumatoid Arthritis!

**DOI:** 10.7759/cureus.11074

**Published:** 2020-10-21

**Authors:** Raffaele A Ruggiero, Qasim Z Iqbal, Zeeshan Zia, Danil Mishiyev, Imran Khalid

**Affiliations:** 1 Internal Medicine, Northwell Health, New York, USA

**Keywords:** interstitial lung disease, complication of rheumatoid arthritis, exertional dyspnea, steroid use, immunosuppressive therapy, rheumatoid arthritis

## Abstract

Rheumatoid arthritis-associated interstitial lung disease (RA-ILD) is a rare extraarticular manifestation of the systemic autoimmune disease rheumatoid arthritis. RA-ILD is one of the leading causes of morbidity and mortality in patients with rheumatoid arthritis. It is more commonly seen in patients with risk factors, which include male sex, severe rheumatoid arthritis, and smoking. The presentation depends on the extent of the underlying pathology. Patients can remain asymptomatic for extended periods of time and progressively develop symptoms such as shortness of breath on exertion and a nonproductive cough. The diagnosis is made using a combination of clinical presentation, pulmonary function testing, and imaging. It is difficult for a practitioner to distinguish the presentation of interstitial lung disease from other causes and RA-ILD. However, it is extremely important to differentiate them due to the differences in management. The treatment usually involves a multidisciplinary team guided approach towards treating the underlying rheumatoid arthritis. Asymptomatic patients are managed by observation, while symptomatic patients are managed with glucocorticoids and immunosuppressants. Accurate and early treatment can lead to an improvement in a patient’s symptoms and significantly improve their quality of life. We present an interesting case of a female with a long-standing history of rheumatoid arthritis not on any treatment presenting to the ED with exertional shortness of breath, dry cough, and abdominal distension.

## Introduction

Rheumatoid arthritis is associated with a wide range of extra-articular manifestations. Patients with pulmonary involvement can present with interstitial lung disease. Rheumatoid arthritis-associated interstitial lung disease (RA-ILD) is, however, a rare extra-articular manifestation of rheumatoid arthritis. RA-ILD even though being rare, is considered as the second most common cause of mortality in patients with rheumatoid arthritis. The patients can have a wide spectrum of clinical symptoms from being asymptomatic to presenting with respiratory failure. The most common presenting complaint in the ED is usually dyspnea on exertion and a dry cough. Management usually requires a multidisciplinary approach, where the ultimate goal is to primarily manage the underlying rheumatoid arthritis. The clinical presentation of other causes of interstitial lung disease is very similar to RA-ILD and this can make it very difficult to differentiate RA-ILD from the other causes. It is crucial, however, to be able to differentiate due to the difference in treatment and overall prognosis of the condition. We present an interesting case of a female with a long-standing history of rheumatoid arthritis not on any treatment presenting to the ED with exertional shortness of breath and abdominal distension.

## Case presentation

A 69-year-old female with a pertinent medical history of congestive heart failure with preserved ejection fraction, liver cirrhosis, seizure disorder, and rheumatoid arthritis (not on treatment) presented to the emergency department due to worsening shortness of breath. This shortness of breath was worse on exertion, was associated with tiredness, dry intermittent cough several times a day lasting for a few minutes, and an increase in her baseline abdominal distension. The dyspnea had no relation to changes in her position, did not wake her up from sleep, and further denied any day time somnolence or snoring at night. She, however, reported that for the past month her symptoms have progressively gotten worse and hence she came to the ED.

**Figure 1:**
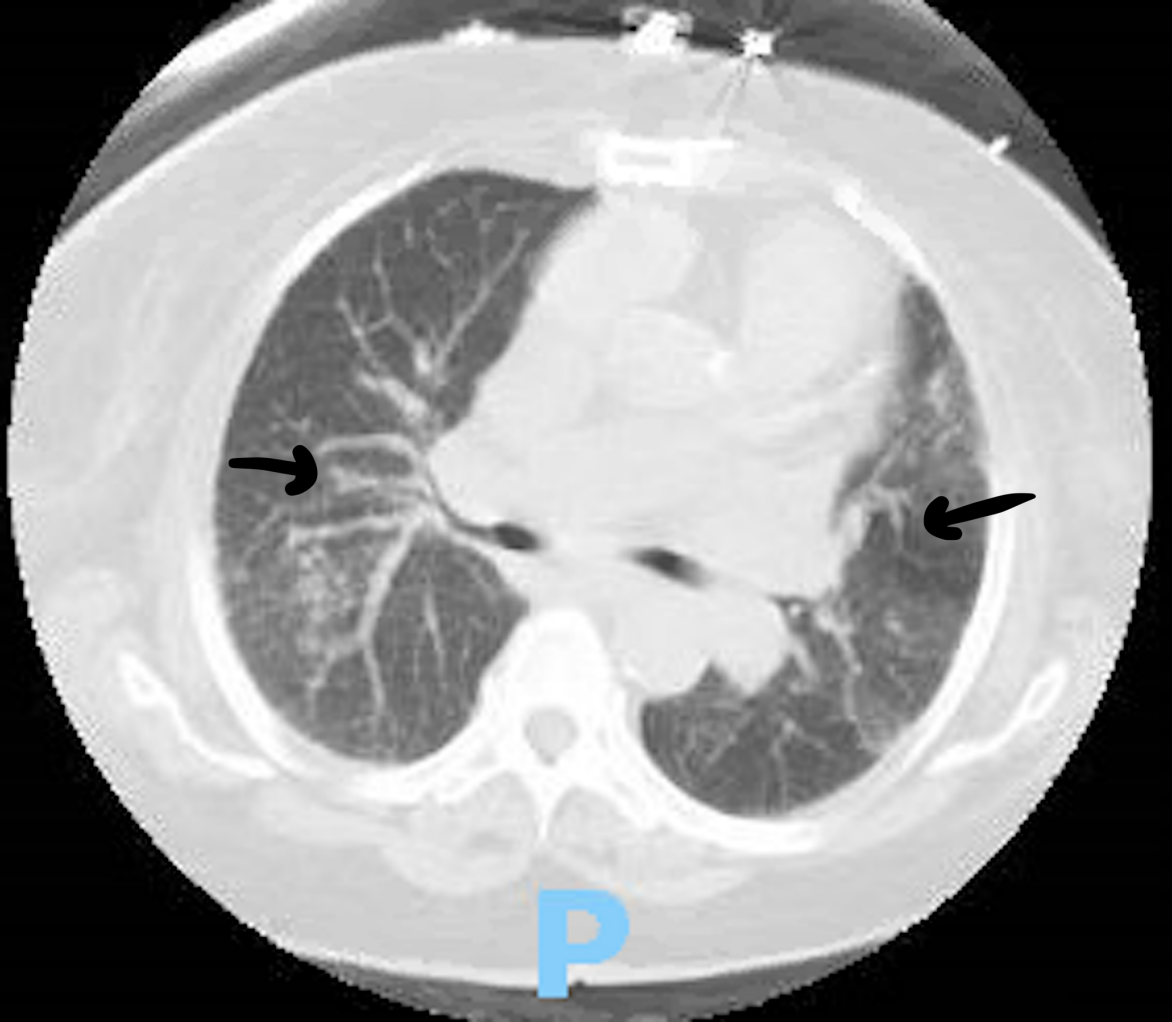
The figure from the CT scan shows bilateral interstitial opacities. Arrows pointing towards characteristic findings of interstitial lung disease.

**Figure 2:**
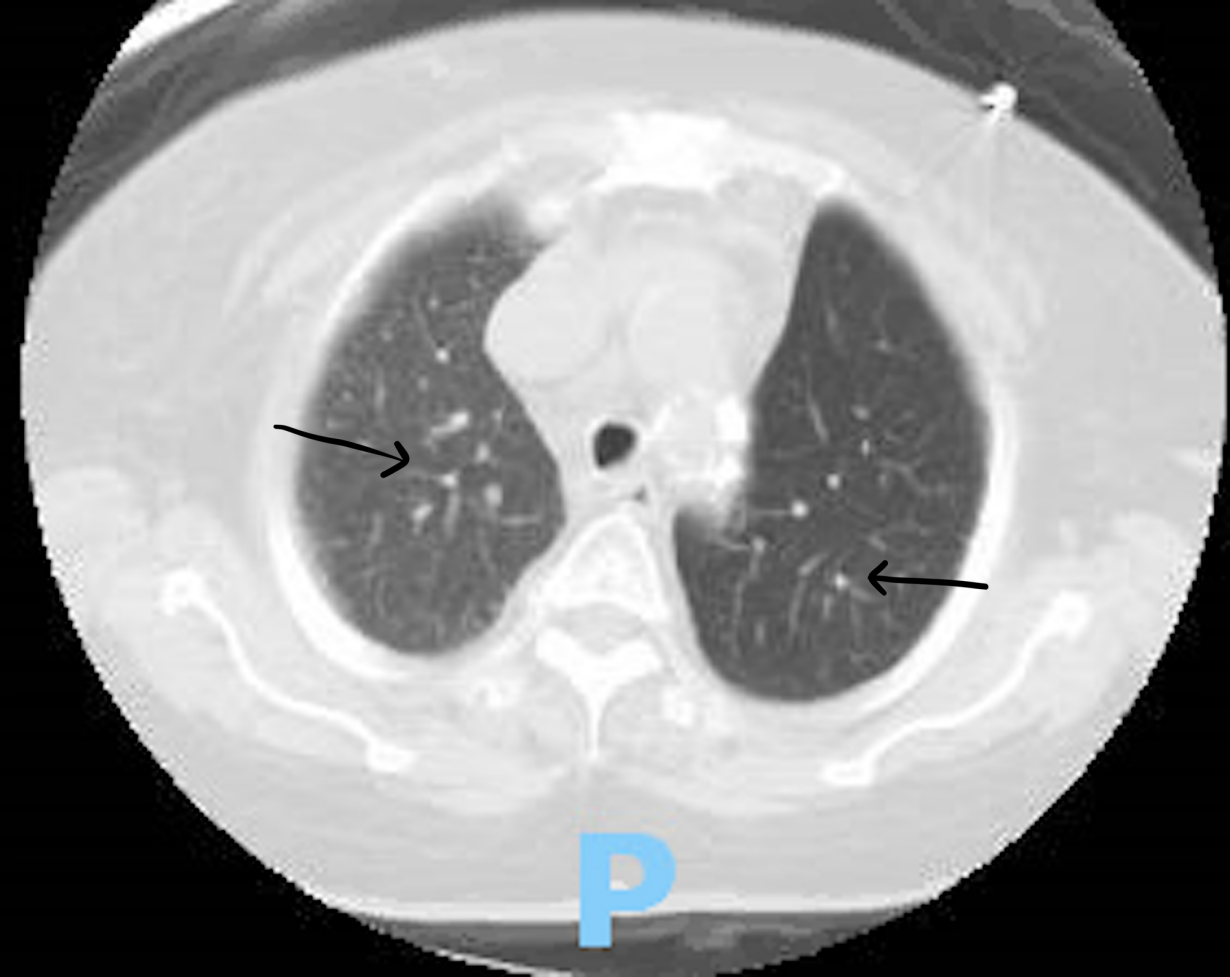
Bilateral interstitial opacities. Arrows pointing towards bilateral interstitial opacities.

On the initial assessment, her vital signs revealed tachycardia and oxygen saturation of 87% for which she was placed on 2 liters of oxygen via nasal cannula. Her physical examination revealed a distended abdomen, crackles were audible at the base of her lungs bilaterally and her lower extremities showed traces of edema bilaterally. Her initial electrocardiogram (EKG) showed sinus tachycardia, her echocardiogram was performed and it revealed normal left ventricular ejection fraction, mild tricuspid regurgitation and severe pulmonary hypertension with the right ventricular systolic pressure estimating to be 66 mmHg. The X-ray done showed bilateral opacities and the CT of her chest (Figure 1 and Figure 2) was suggestive of interstitial lung disease with interstitial thickening, bronchiectasis, and honeycombing. The pulmonary team saw the patient, suggested a rheumatologic workup for the patient to further investigate for a cause of the ILD. The following day, her labs came back and her antinuclear antibody (ANA) was found to be elevated with a ratio of 1:2560, her rheumatoid factor was elevated to >650, and her cyclic citrullinated peptide (CCP) antibody was strongly positive to >250 (Table 1). These results increased our suspicion of rheumatoid arthritis-associated ILD. The rheumatology team also saw the patient and requested laboratory studies to prepare her for a steroid-sparing agent, to be started as an outpatient. The results revealed that her centromere antibody was elevated as well. The patient was started on prednisone 60 mg daily and bronchodilators. The patient's hypoxia improved, she was discharged and she was recommended to follow up with a pulmonologist and a rheumatologist as an outpatient.

**Table 1:** Autoimmune laboratory investigations and their results. TPMT: Thiopurine methyltransferase; ANCA: Antineutrophil cytoplasmic antibodies; ASCA: Anti-Saccharomyces cerevisiae antibodies; CCP: Cyclic citrullinated peptide; ANA: Antinuclear antibody.

Laboratory Studies	Results
C3	110
C4	18
TPMT methodology	29.3
Anti-ribonuclear protein	0.4
SM (Smith) antibody	<0.2
Scleroderma antibodies	<0.2
C-ANCA	Negative
P-ANCA	Negative
Atypical ANCA	Negative
ASCA IgA/IgM	Negative
ANA titer	>1:2560
ANA pattern	Centromere
Centromere antibody	>8
dsDNA antibody	<12
RA Factor	>650
CCP antibody IgG	Strongly positive
Anti-SS-A antibody	<0.2
Anti-SS-B antibody	<0.2

## Discussion

Rheumatoid arthritis is associated with a variety of extra-articular manifestations including lung disease. Interstitial lung disease is one of the pulmonary manifestations of rheumatoid arthritis [1]. Rheumatoid arthritis-associated interstitial lung (RA-ILD) disease and pulmonary complications account for 10 to 20% of mortality in rheumatoid arthritis patients [2]. RA-ILD is not a single type of ILD but rather composed of a spectrum of histopathologic types with different associated patterns of clinical presentation. The most common of which are usual interstitial pneumonia and nonspecific interstitial pneumonia [3,4]. The age of presentation is usually from 50-60, with males being more common than females [5]. Uncontrolled RA disease and cigarette smoking are major risk factors in developing RA-ILD [6,7]. The symptoms of RA-ILD range from being asymptomatic to rapidly fatal acute interstitial pneumonia. Usually, symptoms develop insidiously leading to dyspnea on exertion and a non-productive cough. Later in the disease pulmonary hypertension can develop and eventually patients can develop hypoxic respiratory failure [7]. Physical exam findings tend to be absent in the early stages of the disease. Around 75% of patients eventually develop crackles upon auscultation of the lungs and clubbing can be a rare physical exam finding as well. The diagnosis of the condition is based on a combination of clinical features, pulmonary function testing, high-resolution CT (HRCT) scan, along with the exclusion of other causes. Laboratory findings are useful in the diagnosis. Rheumatoid factor and the anti-CCP antibody are commonly elevated [8]. Lung biopsy is rarely required anymore due to the presence of an HRCT scan which can accurately describe the different pathologic patterns [9]. Lung biopsy these days is only considered for young patients who may be a candidate for a lung transplant. Management requires a multidisciplinary team, geared towards treating the underlying rheumatoid arthritis. Mild disease is monitored, whereas the symptomatic disease is controlled with glucocorticoids and/or immunosuppressive therapy [10]. Smoking cessation is extremely important, as it is the primary preventable risk factor [11]. It is extremely important to differentiate RA-ILD and other causes of ILD when dealing with the management of the disease. RA-ILD management is dependent on controlling rheumatoid arthritis, which in turn can lead to slowing the progression of the disease. The uncontrolled disease can eventually lead to pulmonary hypertension (Group 3) and right heart failure [12]. The exact presentation of RA-ILD depends on the underlying lung pathology and the severity of the disease. This patient’s particular presentation was likely a result of her interstitial lung disease leading to pulmonary hypertension. This in turn most likely led to right-sided heart failure and possibly liver cirrhosis as well. Echocardiogram performed on admission showed evidence of severe pulmonary hypertension, most likely secondary to RA-ILD. The CT of her chest was also suggestive of interstitial lung disease. The laboratory markers for RA such as rheumatoid factor and anti-CCP were elevated, which indicated an active RA disease.

The patients are usually started on initial treatment with glucocorticoids. Steroids are then tapered off and patients are started on a steroid-sparing agent to minimize the side effects of steroids. Furthermore, strict outpatient monitoring will be needed with the patients being monitored with pulmonary function tests (PFTs) and imaging every 6-12 months to track the progression of the disease. General prognosis depends on the histopathologic subtype and the level of impairment of the lungs [13]. Our patient had a nonspecific intestinal pneumonia pattern, which has been associated with a better prognosis compared to other subtypes [14]. Considering that this is one of the main causes of mortality in patients with rheumatoid arthritis, it is important to encourage compliance with the treatment [15].

## Conclusions

We present an interesting case of RA-ILD. It is important to keep it in our differential diagnosis for a patient presenting with worsening shortness of breath especially if the patient has an underlying history of rheumatoid arthritis. The patients usually have symptoms of dry cough and exertional dyspnea. The general prognosis of this disease depends on the histopathologic subtype and the level of impairment of the lungs. Unfortunately, it is one of the main causes of mortality in patients with rheumatoid arthritis, and compliance with the management of the disease is very important.
